# Hypervirulent *Klebsiella pneumoniae* Sequence Type 420 with a Chromosomally Inserted Virulence Plasmid

**DOI:** 10.3390/ijms22179196

**Published:** 2021-08-25

**Authors:** Elias Eger, Stefan E. Heiden, Karsten Becker, Andrea Rau, Katharina Geisenhainer, Evgeny A. Idelevich, Katharina Schaufler

**Affiliations:** 1 Pharmaceutical Microbiology, University of Greifswald, 17489 Greifswald, Germany; elias.eger@uni-greifswald.de (E.E.); stefan.heiden@uni-greifswald.de (S.E.H.); 2 Friedrich Loeffler-Institute of Medical Microbiology, University Medicine Greifswald, 17475 Greifswald, Germany; karsten.becker@med.uni-greifswald.de (K.B.); evgeny.idelevich@med.uni-greifswald.de (E.A.I.); 3 Department of Oral and Maxillofacial Surgery/Plastic Surgery, University Medicine Greifswald, 17475 Greifswald, Germany; andrea.rau@med.uni-greifswald.de (A.R.); katharina.geisenhainer@med.uni-greifswald.de (K.G.); 4 Institute of Medical Microbiology, University Hospital Münster, 48149 Münster, Germany; 5 Institute of Infection Medicine, Christian-Albrecht University and University Medical Center Schleswig-Holstein, 24105 Kiel, Germany

**Keywords:** *K. pneumoniae*, ST420, IS*Kpn74*, chromosomally inserted plasmid

## Abstract

Background: *Klebsiella pneumoniae* causes severe diseases including sepsis, pneumonia and wound infections and is differentiated into hypervirulent (hvKp) and classic (cKp) pathotypes. hvKp isolates are characterized clinically by invasive and multiple site infection and phenotypically in particular through hypermucoviscosity and increased siderophore production, enabled by the presence of the respective virulence genes, which are partly carried on plasmids. Methods: Here, we analyzed two *K. pneumoniae* isolates of a human patient that caused severe multiple site infection. By applying both genomic and phenotypic experiments and combining basic science with clinical approaches, we aimed at characterizing the clinical background as well as the two isolates in-depth. This also included bioinformatics analysis of a chromosomal virulence plasmid integration event. Results: Our genomic analysis revealed that the two isolates were clonal and belonged to sequence type 420, which is not only the first description of this *K. pneumoniae* subtype in Germany but also suggests belonging to the hvKp pathotype. The latter was supported by the clinical appearance and our phenotypic findings revealing increased siderophore production and hypermucoviscosity similar to an archetypical, hypervirulent *K. pneumoniae* strain. In addition, our in-depth bioinformatics analysis suggested the insertion of a hypervirulence plasmid in the bacterial chromosome, mediated by a new IS*5* family sub-group IS*903* insertion sequence designated IS*Kpn74*. Conclusion: Our study contributes not only to the understanding of hvKp and the association between hypervirulence and clinical outcomes but reveals the chromosomal integration of a virulence plasmid, which might lead to tremendous public health implications.

## 1. Background

*Klebsiella pneumoniae* is a leading cause of health care- and community-associated diseases including pneumonia, liver, urinary tract, wound and bloodstream infections, driven by the emergence of antibiotic-resistant classic (cKp) and hypervirulent (hvKp) pathotypes [1,2]. The latter occurs clinically by invasive and multiple site infection including sepsis, meningitis, and liver abscesses [2] and has undergone epidemic spread predominantly in Asia [3]. Convergence of cKp and hvKp isolates with both antibiotic resistance and virulence has also been previously reported [4]. This likely occurs when antibiotic-resistant cKp lineages obtain mobile genetic elements, which carry virulence genes, or when hvKp lineages acquire resistance plasmids [5].

Phenotypically, hvKp isolates appear through hypermucoviscosity, extensive production of siderophores and usual antibiotic susceptibility [3]. Originally, many studies defined hypervirulence as equivalent to a positive ‘string test’, in which a colony stretched 5 mm or longer [3]. This hypermucoviscous phenotype is not very accurately characterized, however, and the definition of hypervirulence remains controversial [6]. Genotypic biomarkers that indicate hypervirulence more reliably include *peg-344* (metabolite transporter, which contributes to virulence in pulmonary infection models [7])*, iroB* (salmochelin)*, iucA* (aerobactin), and *rmpA* and *rmpA2* (modulation of hypermucoviscosity and capsule) [8]. These virulence features are mostly carried on large virulence plasmids and integrative conjugative elements (ICEs) that also encode genes needed for conjugation. ICEs are typically inserted in the bacterial host chromosome and carry genes for integration and excision. Their size ranges from approximately 20 kb to over 500 kb. In contrast, plasmids are known to be extrachromosomal elements that replicate separately and autonomously from the host chromosome. However, several ICEs are able to autonomously replicate similar to plasmids, which makes their classification ambiguous. Both ICEs and conjugative plasmids additionally contain features for processing their genetic content for transfer. Upon stimulation, expression of these genes is induced and the element excises from the host chromosome, replicates and transfers, and integrates elsewhere (from its original source) [9].

In this study, we investigated two *K. pneumoniae* isolates obtained from a human patient to reveal the association between clinical appearance and underlying hypervirulence mechanisms in *Klebsiella* and to determine (i) clonality as well as virulence and resistance genes, (ii) phenotypic virulence, and (iii) a chromosomally inserted virulence plasmid.

## 2. Results and Discussion

The two patient isolates belonged to ST420 and differed in a total of three SNPs only. Since this number is comparable to a recently characterized outbreak of a ST307 *K. pneumoniae* clone that took place in different institutions in north-eastern Germany (on average less than 20 SNPs during the course of the outbreak) [4], we defined them as ‘clonal’. *K. pneumoniae* ST420 has been previously reported from patients with liver abscess and can be likely attributed to the hypervirulent pathotype [10,11]. ST420 predominantly circulates on the Asian continent, in particular in China and Vietnam [12,13]. To the best of our knowledge, this is the first description of *K. pneumoniae* sequence type 420 in Germany.

Kleborate analysis revealed that both genomes carried the “yersiniabactin (*ybt*) lineage *9*” genetic makeup associated with the *K. pneumoniae* integrative conjugative element 3 (ICE*Kp3*) and a resulting yersiniabactin sequence type (YbST) 69. The aerobactin lineage was *iuc 1* with an aerobactin sequence type (AbST) 5. The salmochelin lineage was *iro 1* with a salmochelin sequence type (SmST) 1. Both lineages are linked and normally carried by a non-self-transmissible IncFIB_K_ virulence plasmid type designated KpVP-1 [14]. Capsule (K) and O antigen loci were KL20 (equivalent: K20) and O1v1 with identities of ≥99.98% and ≥98.60%, respectively. Besides the aforementioned salmochelin (*iroBCDN*) and aerobactin (*iucABCD/iutA*) features, *rmpA* and *rmpA2* genes were present as well (Supplementary Table S1).

Note that both isolates carried only a very limited number of antibiotic resistance genes supporting the suggestion that the isolates belong to the hvKp pathotype (Supplementary Table S2). This also corresponded to the results of phenotypic AST, which were identical in isolates from wound and from blood cultures and revealed susceptibility against most of the antimicrobials tested (Supplementary Table S3). Notably, the documented susceptibility patterns of strains that were obtained during the previous infection episodes and that were not available for further investigation showed high phenotypic similarities to the isolates from the present episode. This might suggest possible clonal relation of the patient’s isolates over time and that the episode described here represents a relapse of a recurrent infection. In the absence of the previously isolated strains this, however, cannot be ultimately proven.

The clinical course of infection in our patient revealed typical traits caused by hvKp. First, the infection was severe, as suggested by the invasion of the pathogen into the bloodstream. Second, multiple body sites were affected, which is a hallmark of hvKp [15]. Of note, the patient’s medical history was characterized by multiple *K. pneumoniae* infections. Recurrent courses of disease have rarely been reported for hvKp so far.

**Figure 1:**
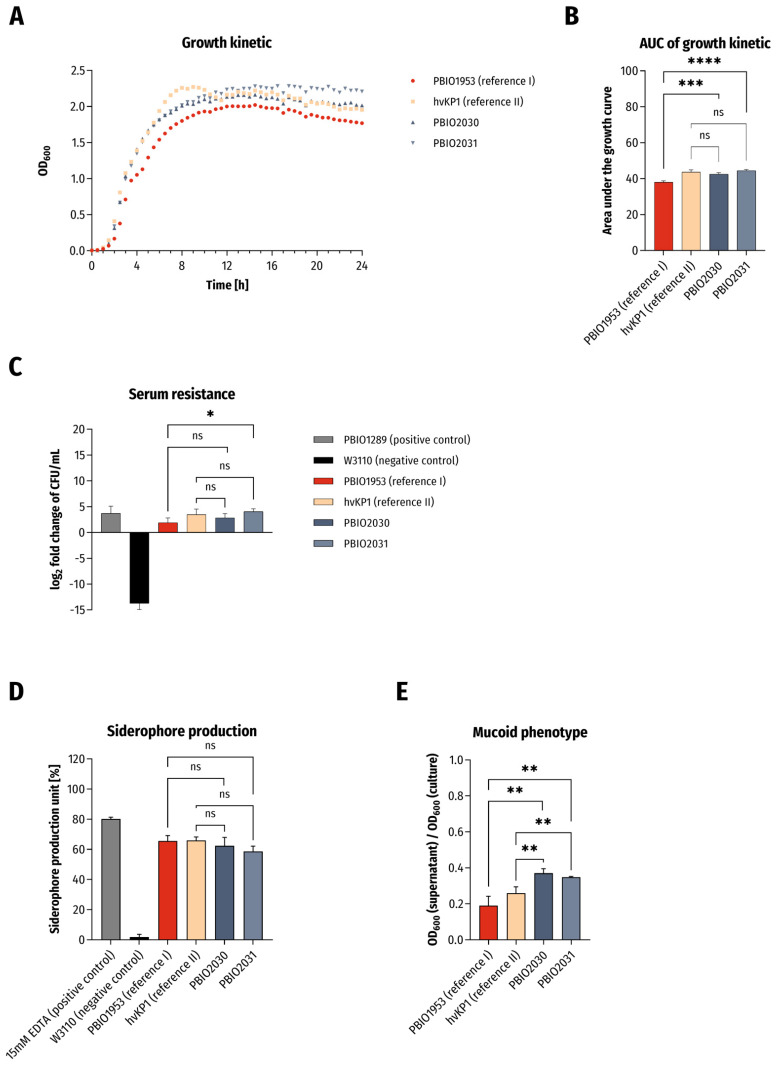
Results of phenotypic experiments. (**A**) Growth kinetics in LB broth and (**B**) statistical comparison of area under the curve (AUC) of growth curves. The results are given as mean values and standard deviation of AUCs. (**C**) Survival in 50% human serum. The results are given as mean values and standard deviation of log_2_ fold change of CFU/mL after 4 h of incubation in serum. (**D**) Siderophore production ability (*n* = 3). The results are given as mean values of siderophore production unit and standard deviation. (**E**) Determination of mucoid phenotype using sedimentation assay (*n* = 3). The results are given as mean ratios of OD_600_ of supernatant after centrifugation at 1000× *g* for 5 min and OD_600_ of the overnight culture and standard deviation. For all results the clinical isolates were compared to PBIO1953 (*K. pneumoniae*, ST307) and hvKP1 (archetypical hypervirulent *K. pneumoniae*) in variance analyses (one-way ANOVA with Dunnett’s multiple comparison post-hoc test). The significance level (*p* value) is as follows: ns not significant, * *p* < 0.05; ** *p* < 0.01; *** *p* < 0.001; **** *p* < 0.0001.

Because of the clinical presentation and genomic outcome, we decided to challenge the isolates in phenotypic experiments associated with hypervirulence (Figure 1). As control strains we included an archetypal hypervirulent *K. pneumoniae* isolate of ST86 (here: “hvKP1”) [16] and the aforementioned “converged” ST307 *K. pneumoniae* recently published by us (PBIO1953) that demonstrated features of hypervirulence and extensive drug resistance [4]. In addition, several internal controls were analyzed and compared (s. Material and Methods section).

First of all, we observed significantly increased growth kinetics of the two ST420 isolates when compared to PBIO1953 (Figure 1A,B; PBIO2030: *p* = 0.0005; PBIO2031: *p* < 0.0001). Similar findings applied to the isolates’ tolerance to human serum (Figure 1C; PBIO2031: *p* = 0.0247). Both ST420 isolates demonstrated siderophore production and hypermucoviscosity in sedimentation and string-test experiments on a minimum level as hvKP1, underlining the hypervirulent character of the two isolates (Figure 1D,E and Table 1). In the case of hypermucoviscosity sedimentation (Figure 1E), they were even significantly better (hvKP1/PBIO2030: *p* = 0.0028, hvKP1/PBIO2031: *p* = 0.0087, PBIO1953/PBIO2030: *p* = 0.001, PBIO1953/PBIO2030: *p* = 0.0021).

The serum tolerance, siderophore production and mucoid phenotypes are likely explained by the presence of genes that encode aerobactin and capsule- and hypermucoviscosity-modulating proteins. The *rmpA* and *rmpA2* regulators of the mucoid phenotype, which increase capsule production, have been shown to increase virulence, possibly by impairing phagocytosis and thus enhancing serum survival [17]. hvKp’s siderophore aerobactin accounts for more than 90% total siderophore production, while others (salmochelin, enterobactin and yersiniabactin) are responsible for the remaining minority. Phenotypic experiments revealed that only aerobactin significantly enhanced strain survival in human serum and mouse infection models, suggesting that aerobactin—in addition to hypermucoviscosity—is one of the major hvKP virulence determinants [18].

It is known that certain *Klebsiella* serotypes—in particular K1, which is represented mostly by ST23, and to a lesser extent K2—are more often associated with hypermucoviscosity and thus severe infection than other capsular types. Another study characterized a serotype K20 strain from a liver abscess, which demonstrated enhanced lethality in mice. However, it did not show hypermucoviscosity despite the complete carriage of a gene set needed for the phenotype [19]. The different mechanisms and their interplay responsible for multiple site infection, hypermucoviscosity, and other virulence features remain limitedly defined [19,20]. Nevertheless, it seems highly likely that the detected geno- and phenotypic characteristics predominantly contributed to the clinical appearance, which includes both the severity of infection (presence of hypervirulence features) and fast recovery upon antibiotic treatment (absence of most antibiotic resistance features).

When investigating the genomic sequences in-depth, we noticed that although the PlasmidFinder results suggested three different Inc types (Supplementary Table S4), we were only able to reconstruct one single plasmid from the sequencing data. In addition, this plasmid had a rather low sequencing depth in the SPAdes graph. While multi-replicon plasmids are not uncommon and the recovered plasmid (96,208 bp) indeed carried IncFII and IncR replicons, this left us with a missing IncHI1B plasmid.

**Figure 2:**
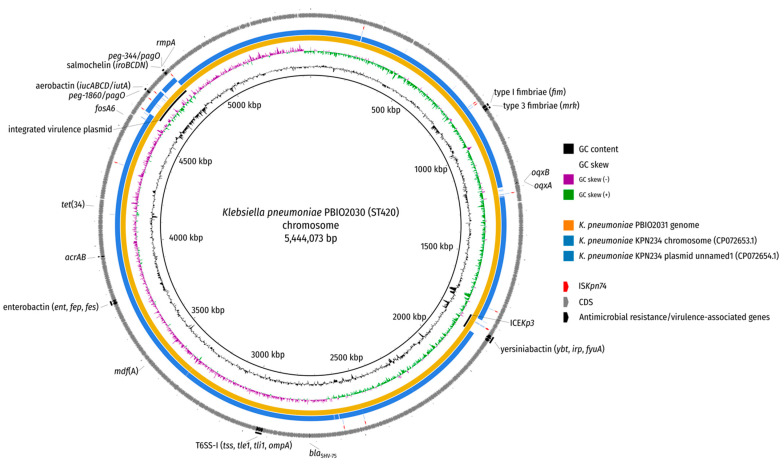
BLAST comparison of *K. pneumoniae* PBIO2030 chromosome. The genome of isolate PBIO2031 (blood culture) and two replicons of a publicly available ST420 isolate (KPN234; NCBI accessions: CP072653.1 (chromosome) and CP072654.1 (plasmid unnamed1) were aligned against the chromosome of PBIO2030 (wound) by BLAST (-task megablast -evalue 1e-10 -dust no). Antimicrobial resistance genes as well as virulence-associated genes are depicted in addition to regions of interest (inserted virulence plasmid, ICE*Kp3*) and the insertion sequence IS*Kpn74*.

**Figure 3:**
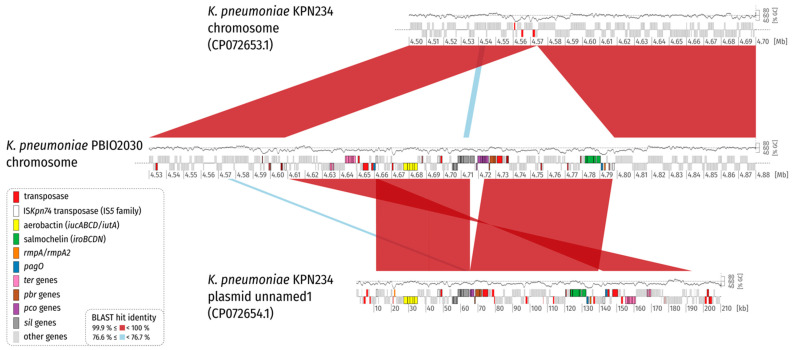
Synteny plot of *K. pneumoniae* PBIO2030 and KPN234 chromosome, and plasmid unnamed1. For clarity, only BLAST hits with a length of at least 1% of the length of the shorter replicon in the comparison are shown (2000 bp and 2102 bp for comparison 1 and 2, respectively). Coding sequences (CDS) are colored according to the legend. A GC plot created by a sliding window (1000 bp, step size = 1 bp) approach is drawn above the replicon backbones. Please note that the GC content is lower for the virulence plasmid and the inserted plasmid region on average. This is also evident in Figure 2. The plot illustrates that a part of the virulence plasmid is missing in its integrated counterpart.

Local BLAST searches located the IncHI1B replicon sequence on the chromosome, which is why the initial SPAdes assembly graph was examined by first identifying putative plasmid nodes through BLAST searches against the nt database and second evaluating the depths of these nodes for irregularities indicative of a possible multiplicity mistake by the Unicycler assembler, which were not detectable. Additional long-read mapping against the chromosome did not reveal any left- or right-clipped regions towards the chromosome–plasmid junctions and also showed an otherwise uniform read depth coverage (Supplementary Figure S1). Note that the hypervirulence-associated *iroBCDN*, *iucABCD/iutA*, *rmpA/A2* and peg* genes were located in this region, which further supports the occurrence of a plasmid integration rather than an ICE (Figure 2 and Figure 3). As final evidence for the integration of the virulence plasmid into the chromosome, we evaluated the PCR results of the left and right junction amplifications using our designed primers. Both amplicons exhibited the expected sizes (~3000 bp and ~4000 bp for primer pairs 1-F + 2-R and 3-F + 4-R, respectively), corroborating the virulence plasmid integration (Supplementary Figure S2). We believe that we are the first to report this type of finding.

When taking a closer look at the left and right flank of the inserted plasmid it became obvious that the inserted region was framed by transposases belonging to the IS*5* family, sub-group IS*903*. Insertion sequences (IS) of this group show typically 950–1150 bp sizes, with inverted repeats (IRs) at their ends, contain transposases with the catalytic residues DDE and duplicate a 9 bp-stretch (direct repeat; DR) during insertion at the target site [21] (Figure 3). Interestingly, the IS elements framing the plasmid integration showed the same direct repeat on the chromosomal side (Figure 2 and Figure 3; Supplementary Table S5). We compared the IS and the encoded transposase with the ISFinder database (https://www-is.biotoul.fr/ (accessed on 12 July 2021) [22]) and noticed that the IS-encoded transposase had an amino acid identity of 93% and 92% when compared to the transposases encoded by IS*903B* and IS*903*, respectively. A BLASTN alignment of the IS nucleotide sequence against the ISFinder database revealed a nucleic acid identity of 96%, 90% and 90% for IS*102*, IS*903B* and IS*903*, respectively. However, the local alignment only considered 1004 bp. A bl2seq (BLAST 2 Sequences) showed 94.52% (IS*102*; 1057 bp), 89.04% (IS*903B*; 1057 bp) and 88.94% (IS*903*; 1057 bp) pairwise identity, corroborating that this IS element is indeed no isoform of a previously described insertion sequence. We requested an attribution number for this IS (IS*Kpn74*). Overall, we identified 14 mobile genetic elements of this type in the chromosome of *K. pneumoniae* PBIO2030 (Supplementary Table S5), with two present in the integrated plasmid region that contained the same single-nucleotide variant (SNV) in the 1056 bp nucleotide sequence.

IS*903* has been previously reported to have the ability to transpose both non-replicatively and replicatively, with the latter allowing the formation of replicon fusion by co-integrate formation [23]. Similarly, transposition can lead to inversions or adjacent deletions that also might have contributed to the plasmid integration described in this study. This is an important evolutionary process, as replicative transposition from a newly integrated plasmid into the recipient chromosome would result in permanent acquisition of all the genetic information from the plasmid via replicon fusion [23].

At this point it remains speculative how exactly and why the virulence plasmid inserted into the chromosome. Answering these questions will be the subject of prospective investigations. It seems possible, however, that chromosomally stabilized virulence features lead to several advantages for the bacterial host. This might include (i) decreased fitness costs compared to isolates that carry plasmid-borne virulence factors, (ii) reduced possibility to lose important virulence features through plasmid segregation [24,25], and (iii) increased possibility to acquire additional, beneficial plasmids (e.g., with resistance genes) [5,26].

## 3. Material and Methods

### 3.1. Strain Origin and Clinical Background

**Figure 4:**
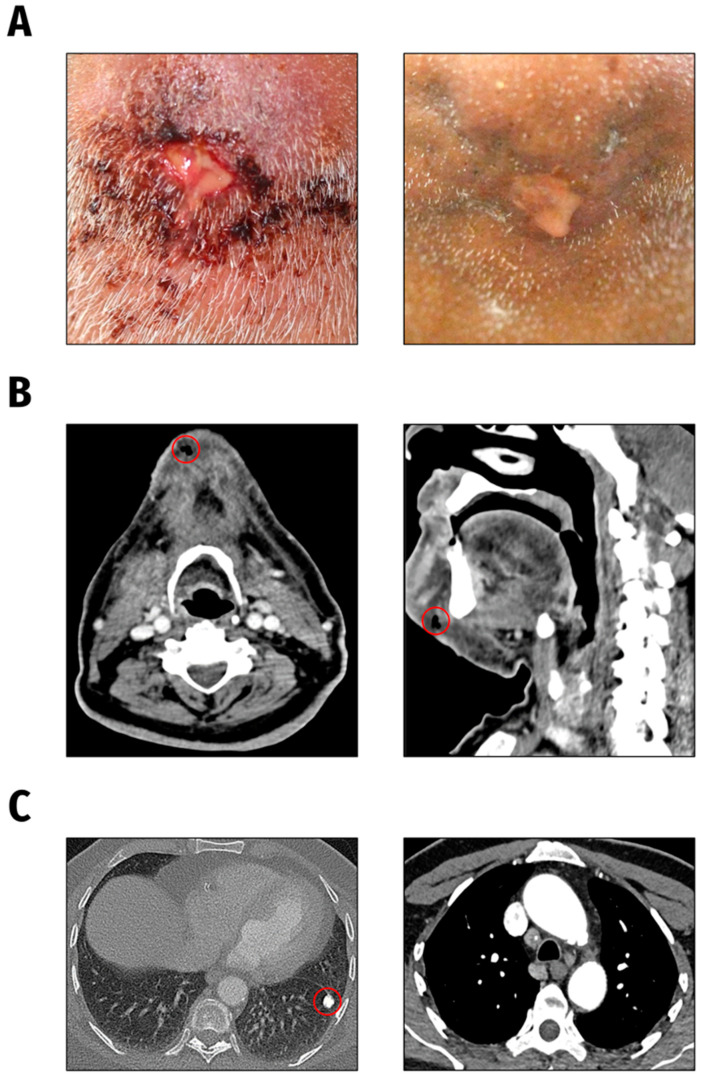
Clinical presentation. (**A**). Submental wound findings at initial presentation (left) and following antibiotic treatment one month later (right). (**B**). CT head and neck findings: subcutaneous air accumulation and circumscribed fluid in the chin region. (**C**). CT chest scan findings: calcified focal lesion in the left lung as well as mediastinal and pulmonary lymphadenopathy.

The *K. pneumoniae* isolates under investigation were obtained from a 66-year-old male patient presented to the Department of Oral and Maxillofacial Surgery/Plastic Surgery, University Medicine Greifswald, Germany in 2021. The patient suffered from severe pain symptoms deriving from an inflammation of the chin region. According to the patient, reddening and swelling of the chin had started one week earlier and he had noticed an increasing putrid secretion from a submental wound (Figure 4A, left). *K. pneumoniae* was cultured from both the chin wound swab and blood culture of the patient.

Previously, *K. pneumoniae* was detected by cultural examination on several occasions. These isolates were obtained one month (from wound swab (nasal abscess)), approximately 1.5 years (from wound swabs and from biopsy tissue (sternal abscess)), and approximately 2.5 years (from wound swab (sinus pilonidalis infection)) prior to the described episode. Unfortunately, these isolates were no longer available for further investigations.

Apart from a chronic cough of unknown cause, the patient stated no further symptoms. Relevant pre-existing conditions included diabetes mellitus, alcohol and nicotine abuse and hypothyroidism. The patient originates from India and lived in Afghanistan for several years.

Intraoral inspection revealed two residual teeth in the lower jaw without any signs of an odontogenic or mucosal infectious focus. Laboratory parameters initially showed moderately elevated infection markers (C-reactive protein and leukocytosis) and hyperglycemia in the context of inadequate diabetes control.

The CT scan of the head and neck showed a circumscribed fluid and air accumulation in the chin region without any osseous affection (Figure 4B). Additionally, in the CT chest scan a rounded calcified pulmonary lesion was detected, highly suspicious of a post-tuberculosis condition (Figure 4C), matching with a positive T-cell interferon-gamma assay (QuantiFERON-TB Gold Plus, Qiagen Biotechnology, Hilden, Germany) result.

A diagnostic biopsy was recommended to the patient, but due to the patient’s incompliance, biopsy taking was not possible. Under systemic antimicrobial therapy with ampicillin/sulbactam and daily antiseptic cleansing of the wound, the initial inflammatory symptoms and submental abscess formation regressed slowly within weeks (Figure 4A, right).

### 3.2. Bacterial Identification and Antimicrobial Susceptibility Testing

Isolates recovered from the wound swab (PBIO2030) and from the blood culture (PBIO2031) were identified as *K. pneumoniae* by matrix-assisted laser desorption ionization-time of flight mass spectrometry (MALDI Biotyper Sirius, Bruker, Bremen, Germany). Phenotypic antimicrobial susceptibility testing (AST) was performed using the Vitek 2 automated system (bioMérieux, Marcy l’Etoile, France).

### 3.3. Phenotypic Analyses

Growth kinetics were assessed by measuring optical densities at λ = 600 nm (OD_600_). Overnight cultures were diluted 1:100 in 5 mL fresh LB broth (Carl Roth GmbH and Co. KG, Karlsruhe, Germany) and incubated at 37 °C and 130 rpm until the OD_600_ reached a 0.5 McFarland standard turbidity. Then, bacterial suspensions were 10-fold diluted and 200 µL of cultures were transferred in triplicates in a 96-well microtiter plate (Nunc™, Thermo Fisher Scientific Inc., Waltham, MA, USA). The OD_600_ was recorded every 30 min by using a microplate reader (FLUOstar Omega, BMG LABTECH GmbH, Ortenberg, Germany) at 37 °C and 200 rpm orbital shaking.

Determination of survival in 50% human serum was performed as described previously [27], with minor modifications. Briefly, overnight cultures were diluted 1:100 in 5 mL fresh LB broth and incubated at 37 °C and 130 rpm until the OD_600_ reached a 0.5 McFarland standard turbidity. Then, bacteria were centrifuged (7500× *g*, 5 min, room temperature) and resuspended in 1 mL of PBS. One hundred microliters of sample were seeded in a 96-well microtiter plate containing 100 µL human serum (US origin, Sigma-Aldrich, St. Louis, MO, USA) per well (resulting in a final concentration of 50% human serum and approximately 10^7^ CFU/mL). Next, 20 µL of each sample were collected and the inoculum size was quantified by plating serial dilutions on LB agar plates incubated at 37 °C overnight. The inoculated microtiter plates were incubated at 37 °C without agitation for 4 h. Thereafter, the number of surviving CFU/mL was determined by plating serial dilutions and following incubation at 37 °C overnight. Controls included in each experiment were the serum-resistant PBIO1289 (*E. coli* ST1159; [28]) and the serum-sensitive W3110 strain.

The quantitative analysis of siderophore secretion was determined using a previously described method [8]. Briefly, bacterial cultures were set to 0.5 McFarland standard turbidity in 0.9% (*w*/*v*) aqueous NaCl solution and 100-fold diluted in iron-chelated M9 minimal salt medium (200 µM 2,2′-Dipyridyl (Carl Roth GmbH and Co. KG, Karlsruhe, Germany) added M9 minimal salt medium (MP Biomedicals, Irvine, CA, USA)) supplemented with 0.3% (*w*/*v*) casamino acids (c-M9-CA, BD, Franklin Lakes, NJ, USA). The strains were grown for 24 h at 37 °C and 130 rpm. Next, 1 mL of bacterial cultures was collected in 1.5 mL tubes (Carl Roth GmbH and Co. KG, Karlsruhe, Germany), centrifuged (4900× *g*, 20 min, room temperature) and 100 µL of siderophore containing supernatant was transferred in triplicates to 96-well microtiter plates containing 100 µL Chromazurol S (CAS) shuttle solution (composited according to [29]). Additionally, fresh media (blank) and 15 mM EDTA (positive control, Carl Roth GmbH and Co. KG, Karlsruhe, Germany) were included. Following incubation in the dark for 30 min at room temperature, the optical density at λ = 630 nm was determined. Secretion of siderophores was expressed as siderophore production units as a percentage calculated according to [30]. A non-siderophore-producing control (W3110) was included.

The hypermucoviscosity sedimentation assay was performed as described previously [31], with some modifications. Again, the bacterial cultures were set to 0.5 McFarland standard turbidity in 0.9% (*w/v*) aqueous NaCl solution and 50 µL of these bacterial suspensions were added to 5 mL of LB broth. Following an incubation period of 24 h at 37 °C and 130 rpm, 1.5 mL of the cultures were collected in 2.0 mL tubes (Carl Roth GmbH and Co. KG, Karlsruhe, Germany) and centrifuged (1000× *g*, 5 min, room temperature). Two hundred microliters of the supernatant as well as 200 µL of the incubated culture were separately transferred each in triplicates to 96-well microtiter plates, and the OD_600_ was measured. The mucoid phenotype was expressed as a ratio of supernatant to total OD_600_. In addition, hypermucoviscosity experiments were performed using the string test. Strings of 5 mm or longer that formed after stretching on the tip of a sterile inoculation loop were defined as positive [32].

All phenotypic experiments were performed with three independent biological replicates.

### 3.4. Genomic Analysis

We generated two whole-genome sequences on an Illumina NextSeq 550 machine in collaboration with the Microbial Genome Sequencing Center (MiGS) in Pittsburgh, PA, USA. In addition, one *K. pneumoniae* isolate (PBIO2030) was long-read sequenced using ONT’s Nanopore system. DNA was extracted using the MasterPure™ DNA Purification Kit for Blood, Version II (Lucigen, Middleton, WI, USA). After quantification and initial quality control, DNA was shipped to MiGS and following library preparation sequenced using 2 × 150 bp paired-end reads.

Raw sequencing reads were adapter-trimmed (k-mer-based trimming using 23-mers down to 11-mers at the right end using the included adapter references; additional trimming by paired read overlap), contaminant-filtered (k-mer-based removal of phiX174 sequences), and quality-trimmed (trimming on both sites for regions with quality < 3; removal of poly G tails ≥ 10 bp; maximum number of Ns after trimming: 0; minimum average quality after trimming: 18; minimum length: 32 bp, filtering reads with entropy below 0.5 to remove low-complexity reads) using BBDuk from BBTools v. 38.90 (http://sourceforge.net/projects/bbmap/ (accessed on 6 July 2021)). Both trimmed reads and raw reads were quality-controlled using FastQC v. 0.11.9 (http://www.bioinformatics.babraham.ac.uk/projects/fastqc/ (accessed on 8 July 2021)). De novo genome assemblies were conducted by employing the assembly pipeline shovill v. 1.1.0 (https://github.com/tseemann/shovill (accessed on 8 July 2021)) in combination with SPAdes v. 3.15.2 [33]. As part of the pipeline, trimmed reads were subsampled to assemble at a maximum coverage of 100×. Besides the polishing step as part of the shovill pipeline, assemblies underwent an additional polishing step. All trimmed reads were mapped back to the contigs using BWA v. 0.7.17 [34]. The obtained SAM/BAM files were sorted and duplicates marked with SAMtools v. 1.12 [35]. Finally, variants were called with Pilon v. 1.23 [36]. The genome for which additional long-read sequencing data were obtained (PBIO2030) was hybrid-assembled with Unicycler v. 0.4.9 [37] in combination with SPAdes v. 3.13.0 [33]. Genome quality and completeness were assessed with CheckM v. 1.1.3 [38]. We used Prokka v. 1.14.6 [39] to annotate draft and finished genomes automatically.

The in silico multi-locus sequence typing (MLST) and antibiotic resistance/virulence gene detection were carried out using mlst v. 2.19.0 (https://github.com/tseemann/mlst (accessed on 09 July 2021)) and ABRicate v. 1.0.0 (https://github.com/tseemann/abricate (accessed on 09 July 2021)), respectively. Both tools rely on third-party public databases (e.g., PubMLST [40], VFDB [41], ResFinder [42], PlasmidFinder [43], BacMet [44]). To visualize genome content, BRIG v. 0.95-dev.0004 [45] and NCBI BLAST v. 2.11.0+ [46] were employed by aligning isolate contigs against the closed reference of PBIO2030. For in-depth typing of yersiniabactin, aerobactin, salmochelin, K locus, and O locus, we used Kleborate v. 1.0.0 with Kaptive [47,48]. A synteny plot comparing the region harboring the chromosomally integrated plasmid with two replicons (chromosome and plasmid) of a publicly available ST420 isolate (*K. pneumoniae* KPN234; NCBI accessions CP072653.1 and CP072654.1) was created with genoPlotR v. 0.8.9 [49].

### 3.5. Validity of Plasmid Integration (In Silico and PCR Analysis)

The validity of the chromosomal integration of a virulence plasmid was initially checked by investigating the depth of nodes in the SPAdes assembly graph with Bandage v. 0.8.1 [50]. Additionally, after hybrid assembly, the long-reads were mapped to the resulting replicons with minimap2 v. 2.17 [51] and visualized with Tablet v. 1.21.02.08 [52].

To further corroborate this integration, two specific primer pairs for amplifying the left and right junction of chromosome and plasmid were derived. PBIO2030_1-F (5′-GAGCGTCAGAGGTATCGTCG-3′) and PBIO2030_2-R (5′-CCAAACAGCACACATCCAGC-3′) as well as PBIO2030_3-F (5′-ACGGGTTTACAGCGATCAGG-3′) and PBIO2030_4-R (5′-AGGTGAGTTCATCAGGCAGC-3′) were used to generate amplicons with expected sizes of 2860 bp and 3939 bp, respectively. A third amplification using primers PBIO2030_1-F and PBIO2030_4-R was conducted, which should yield amplicons of sizes 3492 bp and 2427 bp if no plasmid or even no IS insertion was present in the chromosome, respectively. The polymerase chain reactions (PCR) were performed in 50 µL volumes, each containing 5 µL of template DNA, 0.5 µM of each primer and PCR Master Mix (Thermo Fisher Scientific Inc., Waltham, MA, USA) with the following conditions: initial denaturation at 95 °C for 3 min followed by 30 cycles each of 95 °C for 30 s (denaturation), 59 °C for 30 s (annealing) and 72 °C for 3 min (elongation), and a final extension at 72 °C for 10 min. Five microliters of each PCR product was electrophoresed on a 1% agarose gel (Biozym, Hessian Oldendorf, Germany) supplemented with GelRed (Biotium, Fremont, CA, USA) and visualized by UV light.

## 4. Conclusions

Here, we report the characteristics of two clonal ST420 *K. pneumoniae* isolates that caused a multiple site infection in a human patient from Germany. Their underlying hypervirulence-associated features likely contributed to the clinical appearance and outcome. In addition, we revealed the presence of a chromosomally inserted virulence plasmid with potentially tremendous implications for public health; for example, the emergence of hvKp pathotypes that additionally acquire antibiotic resistance plasmids.

## Figures and Tables

**Table 1:** Results of string test. A positive result is given at a string length ≥ 5 mm.

Strain (Origin, Purpose)	String Test Result
W3110 (*E. coli*; negative control)	Negative
ATCC700603 (*K. pneumoniae*; negative control)	Negative
PBIO1953 (reference I)	Negative
hvKP1 (reference II)	Positive
PBIO2030 (wound)	Positive
PBIO2031 (blood culture)	Positive

## Data Availability

The sequence data for this study have been deposited in the European Nucleotide Archive (ENA) at EMBL-EBI under accession number PRJEB46059 (https://www.ebi.ac.uk/ena/browser/view/PRJEB46059 (accessed on 9 August 2021)). All additional data can be found in this article and the supplements or upon request from the corresponding author.

## Supplementary Materials

The following are available online at https://www.mdpi.com/article/10.3390/ijms22179196/s1. Figure S1: Visualization of mapping long-reads to the hybrid assembly of *K. pneumoniae* PBIO2030, Figure S2: The representative electrophoresis gel picture of PCR products supports the chromosomal insertion of a virulence plasmid, Table S1: Virulence factor database (VFDB) results, Table S2: ResFinder database results, Table S3: Results of phenotypic antimicrobial susceptibility testing, Table S4: PlasmidFinder database results, Table S5: IS*Kpn74* occurrence in the *K. pneumoniae* PBIO2030 chromosome.

## Abbreviations

AbST	aerobactin sequence type
AST	antimicrobial susceptibility testing
bp	base pairs
CAS	Chromazurol S
CFU	colony-forming unit
cKp	classic *K. pneumoniae*
DR	direct repeat
hvKp	hypervirulent *K. pneumoniae*
ICE	integrative conjugative element
Inc	incompatibility group
IR	inverted repeat
IS	insertion sequence
kb	kilo base pairs
µL	microliter
mL	milliliter
MLST	multi-locus sequence typing
OD	optical density
PBS	phosphate-buffered saline
PCR	polymerase chain reaction
SmST	salmochelin sequence type
SNP	single-nucleotide polymorphism
SNV	single-nucleotide variant
ST	sequence type
YbST	yersiniabactin sequence type
