# Supplementary material for: Hypervirulent Klebsiella pneumoniae Sequence Type 420 with a Chromosomally Inserted Virulence Plasmid
Source: Int J Mol Sci. 2021 Aug 25;22(17):9196. doi: 10.3390/ijms22179196 (PMC8431375; doi:10.3390/ijms22179196)
Supplement: Supplementary file 1 [file ijms-22-09196-s001.zip › Suppl. Figures.pdf]

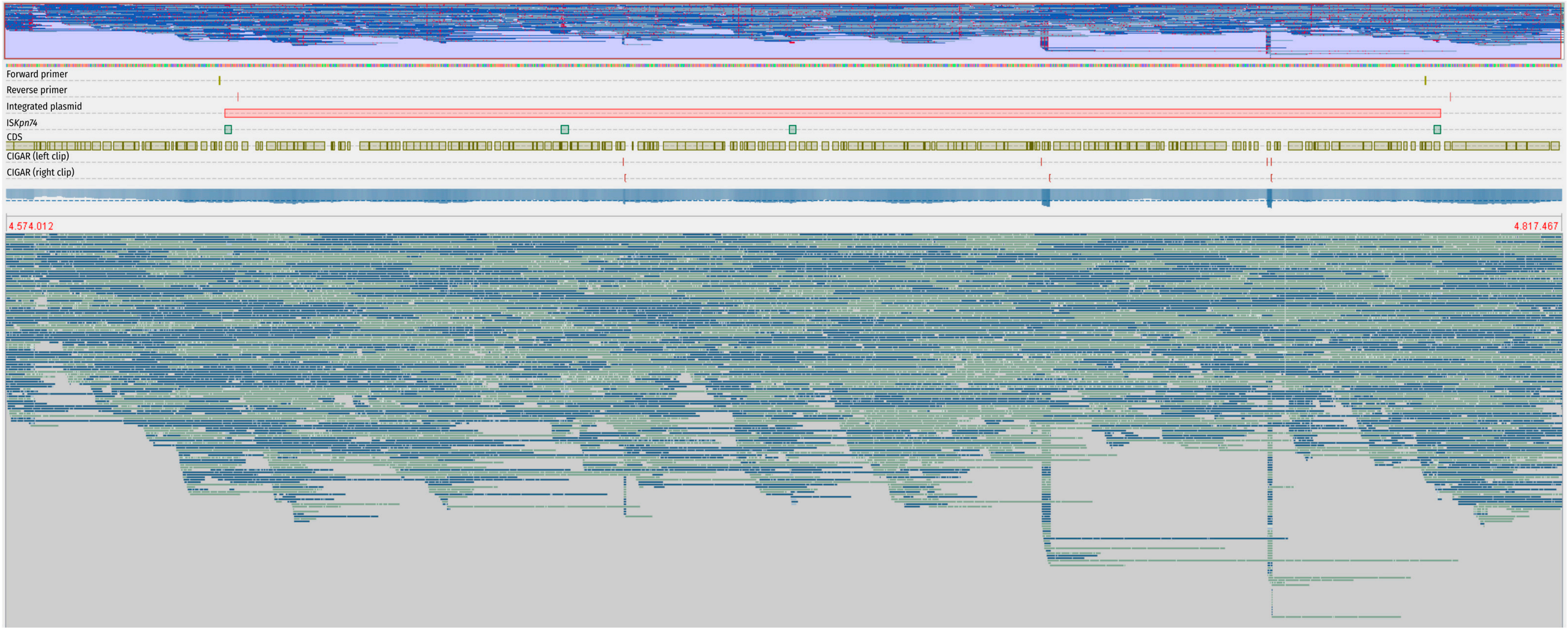

Supplementary Figure S1: Visualization of mapping long-reads to the hybrid assembly of *K. pneumoniae* PBIO2030. Shown is the region containing the integrated virulence plasmid. The blue colored histogram indicates the mapping coverage. Reads mapping in the forward direction are colored green, whereas reverse reads are colored blue. On the top, sequence features like CDS or the positions of forward (PBIO2003_1-F and PBIO2003_3-F) and reverse primer binding sites (PBIO2003_2-R and PBIO2003_4-R) at the left and right junction used for PCR analysis of plasmid integration are shown (Supplementary Figure 2). Additionally, the integrated plasmid as well as positions of IS*Kpn74* are indicated. It is to be observed that there is no indication of read clipping at the chromosome-plasmid junctions and the overall sequence coverage is uniform.

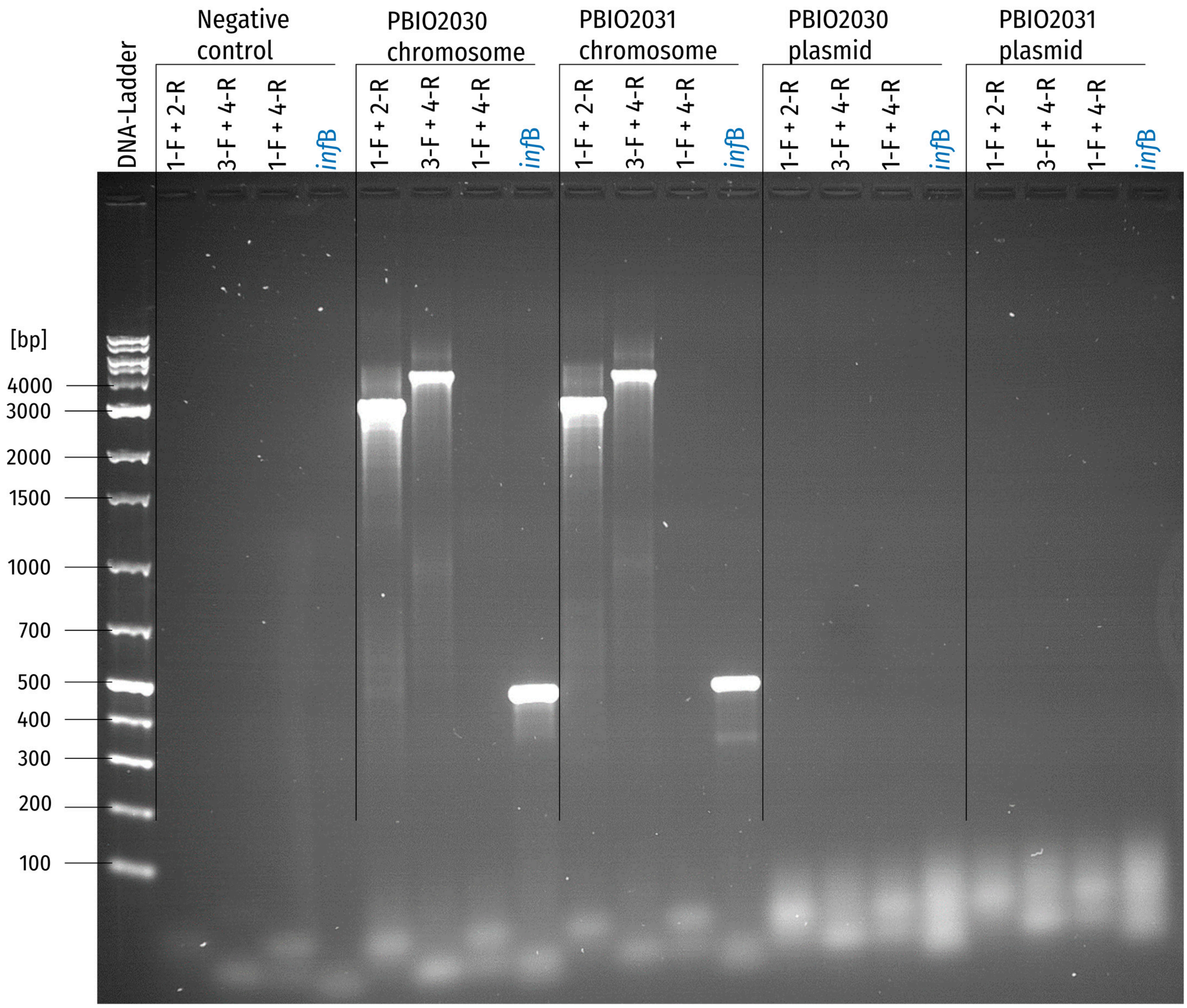

Supplementary Figure S2: The representative electrophoresis gel picture of PCR products supports the chromosomal integration of a virulence plasmid. Two specific primer pairs were used for amplification of left and right junction sequences of chromosome and integrated plasmid. The expected amplicons were sized 2,860 bp (1-F + 2-R, left junction) and 3,939 bp (3-F + 4-R, right junction), respectively. Additionally, the primers 1-F and 4-R were combined to exclude the opportunity that no plasmid or even no IS insertion was present in the chromosome. For comparative reasons, plasmid and genomic DNA were used as template. To ensure that chromosomal DNA is only present when using genomic DNA, a primer pair for the housekeeping gene *inf*B (translation initiation factor 2, amplicon size 462 bp) was included. The negative control had water substituted for template DNA.
