# Supplementary figures and images for: Hypervirulent Klebsiella pneumoniae Sequence Type 420 with a Chromosomally Inserted Virulence Plasmid
Source: Int J Mol Sci. 2021 Aug 25;22(17):9196. doi: 10.3390/ijms22179196 (PMC8431375; doi:10.3390/ijms22179196)

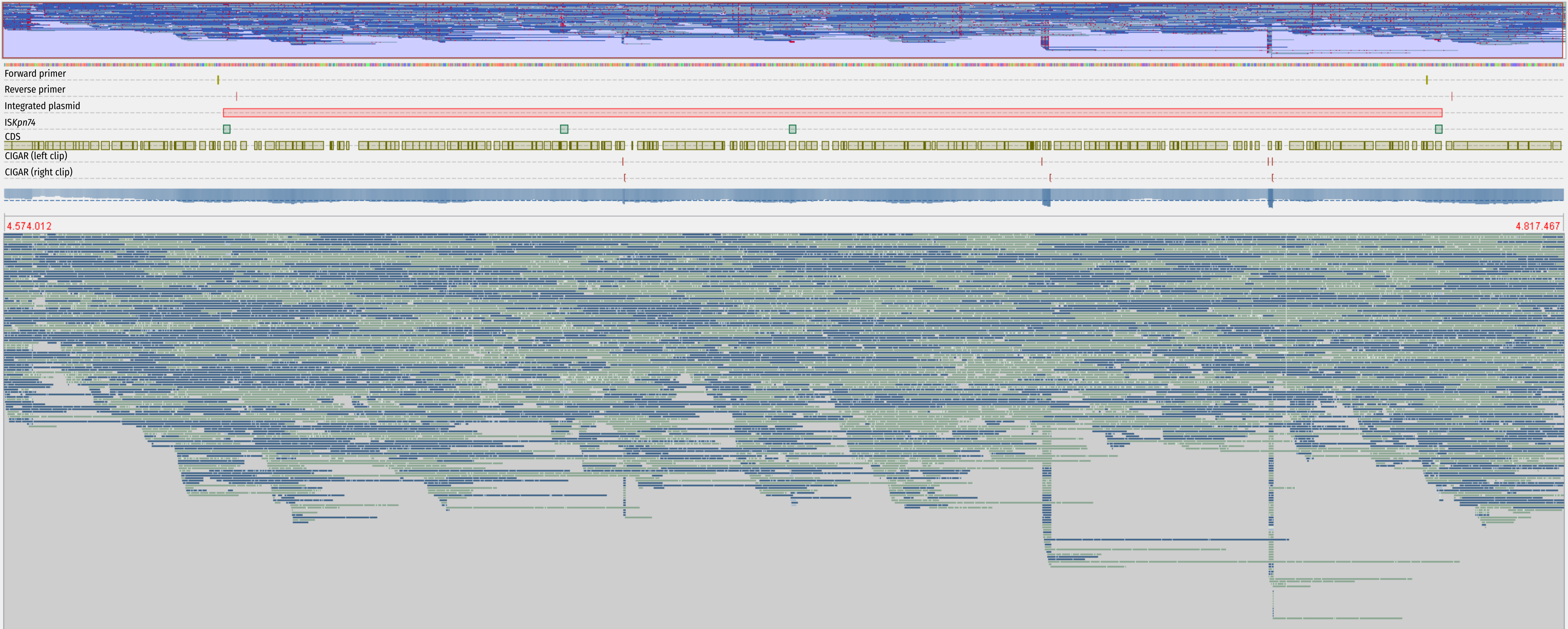

Supplement: Supplementary file 1 [file ijms-22-09196-s001.zip › Suppl. Fig. 1.png]

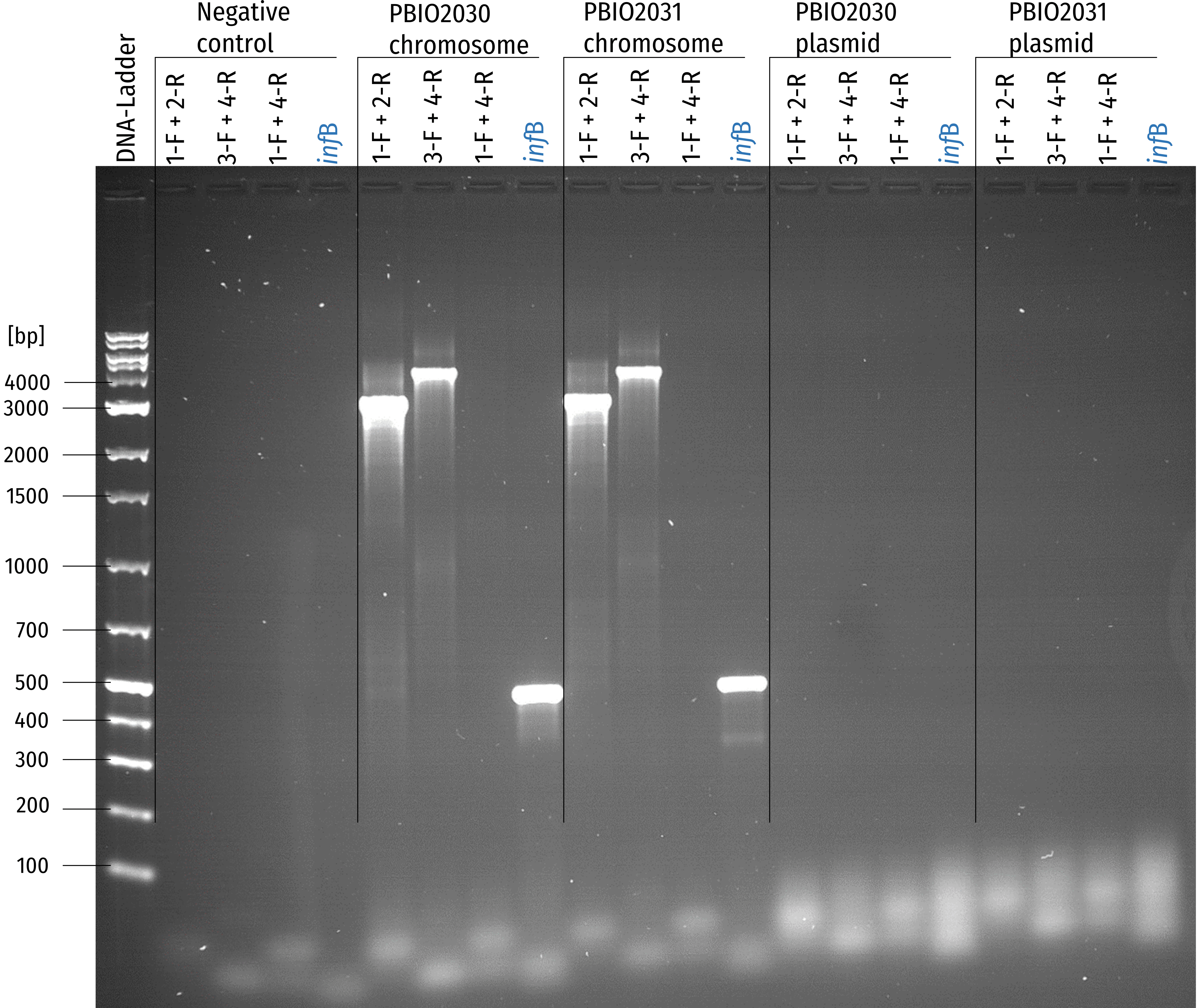

Supplement: Supplementary file 1 [file ijms-22-09196-s001.zip › Suppl. Fig. 2.png]
